# Serum Omentin-1 as a Disease Activity Marker for Crohn's Disease

**DOI:** 10.1155/2014/162517

**Published:** 2014-02-11

**Authors:** Yan Lu, Li Zhou, Lifeng Liu, Yan Feng, Li Lu, Xiaoyan Ren, Xinqian Dong, Weiwei Sang

**Affiliations:** ^1^Department of Gastroenterology, Liaocheng People's Hospital, Liaocheng 252000, China; ^2^Department of Pathology, Liaocheng Second People's Hospital, Linqing 252600, China; ^3^Department of Radiology, Binzhou Medical College Hospital, Binzhou 256603, China; ^4^Department of Pharmacy, Liaocheng International Peace Hospital, Liaocheng 252000, China; ^5^Central Laboratory, Liaocheng People's Hospital, Liaocheng 252000, China; ^6^Department of Pathology, Liaocheng People's Hospital, Liaocheng 252000, China

## Abstract

*Background and Aim*. It remains challenging to determine the inflammatory activity in Crohn's disease (CD) for lack of specific laboratory markers. Recent studies suggest that serum omentin-1 is associated with inflammatory response. We aimed to assess the potential of serum omentin-1 as a marker of disease activity in CD patients. *Methods*. Serum omentin-1 concentrations were determined by enzyme-linked immunosorbent assay (ELISA) in patients with CD (*n* = 240), functional gastrointestinal disorders (FGDs, *n* = 120), and healthy controls (HC, *n* = 60) and evaluated for correlation with disease activity. Expression of omentin-1 in colonic tissues from patients with CD was also analyzed by real-time PCR and Western blotting. Serum omentin-1 levels as an activity index were evaluated using a receiver operating characteristic (ROC) curve. *Results*. Serum omentin-1 concentrations were significantly decreased in active CD patients compared with patients in remission, FGDs, and HC (all *P* < 0.001). Expression of omentin-1 was decreased at mRNA and protein levels in inflamed colonic tissues in active CD than that in noninflamed colonic tissues. Serum omentin-1 levels were negatively correlated with disease activity in CD, better than C-reactive protein (CRP). *Conclusion*. Our results indicate that serum and colonic omentin-1 expressions are decreased in active CD patients. The correlation of serum omentin-1 with disease activity in CD is superior to that of CRP. Serum omentin-1 is a potential marker for CD disease activity.

## 1. Introduction

Although genetic, infectious, and immunological factors may play roles in Crohn's disease (CD), the etiology of CD remains idiopathic. In the course of CD, it is challenging to differentiate between CD flare and inflammation-unrelated intestinal distress [1]. Moreover, CD frequently presented with extraintestinal manifestations, which should be interpreted both clinically and biologically. In current clinical practice, Crohn's Disease Activity Index (CDAI) is still the most commonly used method to assess the inflammatory activity in CD [2]. However, a recent report demonstrates that the CDAI does not discriminate patients with symptoms due to active Crohn's from patients with irritable bowel syndrome [3]. Commonly used inflammatory markers, such as C-reactive protein (CRP) and the erythrocyte sedimentation rate (ESR) also have obvious limitations, as these parameters lack specificity for CD and can be affected by a number of concurrent factors [4].

Omentin is a novel visceral fat-specific adipokine encoded by two genes (1 and 2), mainly expressed in visceral omental adipose tissue [5]. Omentin-1 has been shown to be the major circulating isoform in human serum [6]. Serum omentin-1 level has been reported to be significantly downregulated in obesity, polycystic ovary syndrome, diabetes, and coronary artery disease [7–10]. A recent study concludes that omentin may play an anti-inflammatory role by preventing the tumor necrosis factor alpha (TNF-*α*)-induced COX-2 expression in vascular endothelial cells [11]. Considering that inflammation plays a crucial role in the mechanism of CD, the aim of our study was to evaluate the potential of serum factor as a marker of disease activity of CD.

## 2. Materials and Methods

### 2.1. Study Design

The study was a prospective, single-centered study performed over a 20-month period. Diagnosis of CD was based on conventional clinical, radiological, endoscopic, and histopathological criteria [12]. Sera and surgical or biopsy samples were obtained from patients with CD (*n* = 240) from Liaocheng People's Hospital. Serum was collected from all the patients enrolled before operation. The controls were the patients with age- and gender-matched functional gastrointestinal disorders (FGDs, *n* = 120) using the Rome III criteria [13] and healthy volunteers (*n* = 60).

Clinical activities were determined using Crohn's Disease Activity Index (CDAI) [2]. A CDAI ≥ 150 was defined as the active phase of the disease and a CDAI < 150 as remission. Informed consent was obtained from all participants and all studies involving human subjects were approved by the Institutional Review Boards of Liaocheng People's Hospital.

### 2.2. Quantification of Serum Omentin-1

Human serum omentin-1 levels were quantitated by human omentin-1 assay kit (Aviscera Bioscience, USA). These enzyme-linked immunosorbent (ELISA) assays were performed in duplicate following the manufacturer's guidelines.

### 2.3. Quantitative Reverse-Transcription Polymerase Chain Reaction (qRT-PCR)

For the quantification of mRNA levels of omentin-1 by qRT-PCR, frozen colon tissue samples were analyzed using the QuantiTect SYBR Green RT-PCR kit (QIAGEN) according to the instructions. Levels of colon omentin-1 and control GAPDH levels were determined by the 7900HT Real-time PCR system (Applied Biosystems) using specific primers: omentin-1 forward 5′-ACGTGCCCAATAAGTCCCC-3′; reverse 5′-CCGTTGTCAGTCCAACACTTTC-3′ and GAPDH forward 5′-AATGGACAACTGGTCGTGGAC-3′; reverse 5′-CCCTCCAGGGGATCTGTTTG-3′.

### 2.4. Western Blot

Frozen colon tissue samples were lysed in RIPA buffer (Qiagen) followed by centrifugation (12,000 rpm, 4°C, 15 minutes), after which the supernatants were stored at −80°C until use. Extracted proteins were subjected to sodium dodecyl sulfate-polyacrylamide gel electrophoresis (SDS-PAGE) and transferred to polyvinylidene difluoride (PVDF) membranes. The membrane was blocked with Tris-buffered saline containing 0.1% Tween 20 (pH 7.6) for 1 hour at room temperature. Subsequently, the PVDF membrane was immunoblotted overnight at 4°C with the primary antibody solution (1 : 1,000). After washing twice with TBST, the membrane was incubated with horseradish peroxidase-labeled secondary goat anti-mouse IgG antibody (Santa Cruz Biotechnology) for 1 hour at room temperature and thereafter washed three times with TBST. The final detection was performed with enhanced chemiluminescence Western blotting reagents (GE Healthcare Bio-Sciences Corp.) and the membranes were exposed to Lumi-Film Chemiluminescent Detection Film (Roche Applied Science).

Loading differences were normalized by using the housekeeping control GAPDH. The primary antibodies used in this study included anti-omentin-1 and anti-GAPDH (both from Santa Cruz Biotechnology).

### 2.5. Statistical Analysis

The Mann-Whitney *U*-test or one-way analysis of variance (ANOVA) was used for statistical analyses. Two-tailed Student's *t*-test was used for significant differences in omentin-1 expression between identical patients with CD in active and remission disease stage. Pearson's test was used to analyze the relationship between omentin-1 and CRP or CDAI. SPSS17.0 software was used to draw receiver operating characteristic (ROC) curves and estimate the area under the ROC curve (AUC). *P* < 0.05 was considered significant.

## 3. Results 

### 3.1. Serum Omentin-1 Levels Are Decreased in Active CD Patients

**Figure 1:**
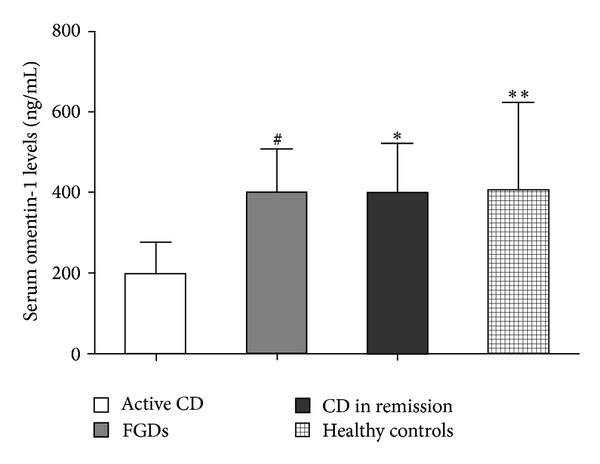
Serum omentin-1 concentrations in the healthy controls (HC, *n* = 60), functional gastrointestinal disorders (FGDs, *n* = 120), active Crohn's disease (CD, *n* = 112), and CD in remission (*n* = 128). ^#^ *P* < 0.01 versus active CD, *P < 0.01 versus active CD, **P < 0.01 versus active CD.

Detailed patient characteristics are presented in Tables 1 and 2. We quantified serum omentin-1 concentrations by ELISA using sera from patients with CD. Serum omentin-1 concentrations were significantly decreased in the active CD patients compared with control FGDs (201.29 ± 76.65 ng/mL versus 401.40 ± 108.36 ng/mL; *P* < 0.0001) (Figure 1). There was also a significant difference between omentin-1 serum levels in patients with active CD compared with CD in remission (201.29 ± 76.65 ng/mL versus 404.61 ± 121.02 ng/mL; *P* < 0.0001) (Figure 1). To further confirm these results, we also determined the serum omentin-1 levels in healthy controls (*n* = 60). The serum omentin-1 levels were also significantly reduced in active CD patients compared with healthy controls (201.29 ± 76.65 ng/mL versus 409.40 ± 215.65 ng/mL; *P* < 0.0001) (Figure 1). In addition, we also compared the omentin-1 levels between CD patients with complications and those without complications. We did not find a significant difference between them (298.71 ± 98.87 ng/mL versus 306.26 ± 154.30 ng/mL; *P* > 0.05). These results suggest that serum omentin-1 levels are decreased in acute intestinal inflammation.

### 3.2. Serum Omentin-1 Levels Are Correlated with Disease Activity in CD Patients

**Figure 2:**
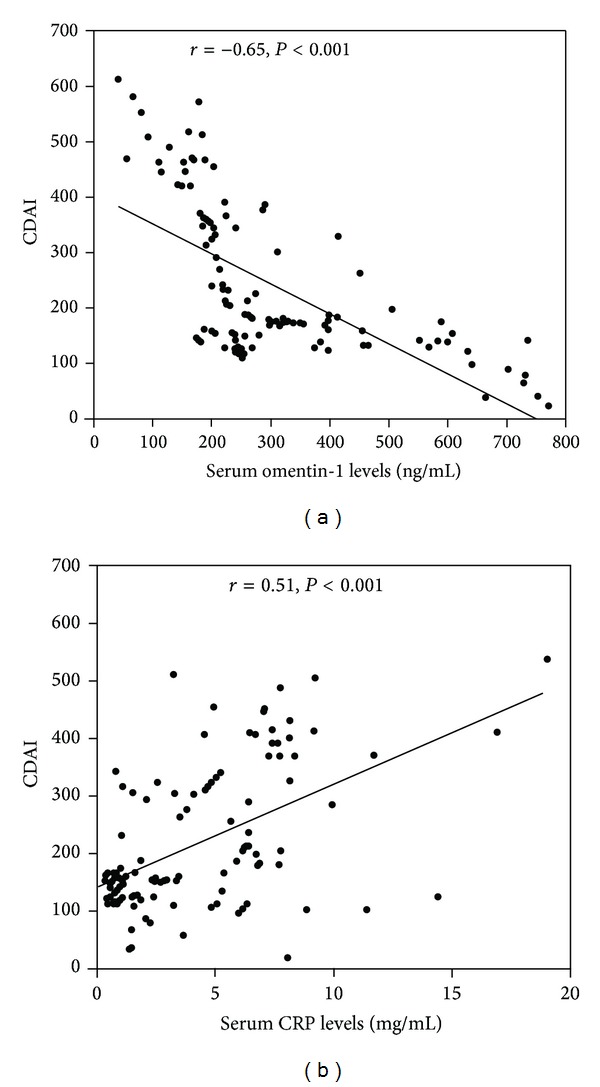
Serum omentin-1 levels are correlated with disease activity in Crohn's disease (CD) patients. A negative correlation was observed between serum omentin-1 level and CDAI (*r* = −0.65, *P* < 0.0001) (a). There was a positive correlation observed between CRP and CDAI (*r* = 0.51, *P* < 0.0001) (b).

We investigated the correlation between serum omentin-1 levels and disease activity (CDAI) in CD patients. A negative correlation was observed between serum omentin-1 level and CDAI (*r* = −0.65, *P* < 0.0001) (Figure 2(a)). There was a positive correlation observed between CRP and CDAI (*r* = 0.51, *P* < 0.0001) (Figure 2(b)). While serum omentin-1 levels were inversely correlated with CRP levels in patients with CD (*r* = −0.62, *P* < 0.0001), such a correlation was not found when a CRP-negative subgroup (CRP cutoff value <3.4 mg/mL) was analyzed (*r* = 0.07, *P* = 0.59). In this CRP-negative group, serum omentin-1 levels remained significantly correlated with CDAI (*r* = −0.52, *P* = 0.0013). However, in this subgroup, significant correlation was not found between CRP and CDAI (*r* = 0.093, *P* = 0.63). These findings account for a better correlation of CDAI with omentin-1 than that with CRP.

### 3.3. Expression of Omentin-1 Was Decreased in Inflamed CD Colons

**Figure 3:**
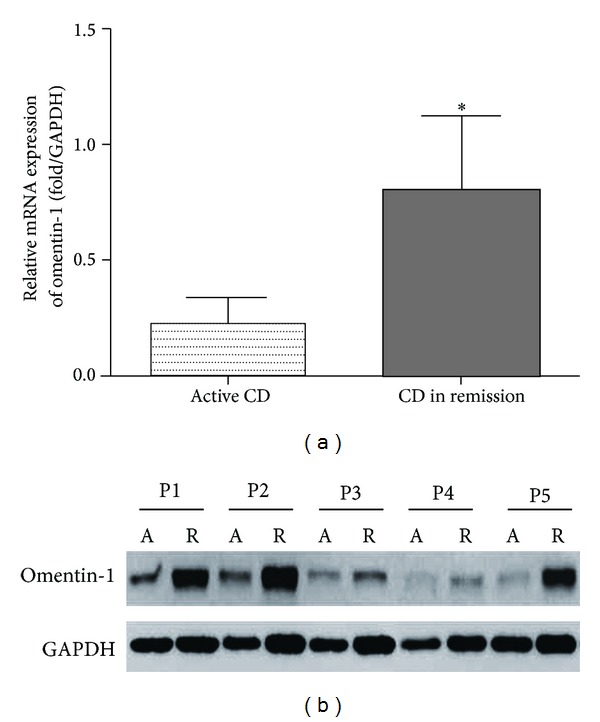
Expression of omentin-1 was decreased in inflamed Crohn's disease (CD) colons. qRT-PCR results showed that omentin-1 expression in colon tissues was decreased in inflamed sites of active CD patients compared with noninflamed colon tissues (a). Western blot analysis confirmed the lower omentin-1 in inflamed colon tissues than that in noninflamed sites (b).

Furthermore, to investigate whether local inflammatory sites in patients with CD are a potential source of decreased serum omentin-1, we detected the expression of omentin-1 in the colon by qRT-PCR and western blot in inflamed and noninflamed sites of surgically resected full-thickness colon specimens from patients with CD. qRT-PCR results showed that omentin-1 expression in colon tissues was decreased in inflamed sites of active CD patients compared with noninflamed colon tissues (Figure 3(a)). Furthermore, western blot analysis confirmed the lower expression of omentin-1 in inflamed colon tissues than that in noninflamed sites (Figure 3(b)).

### 3.4. Evaluation of Serum Omentin-1 Levels as an Activity Index in CD Patients

**Figure 4:**
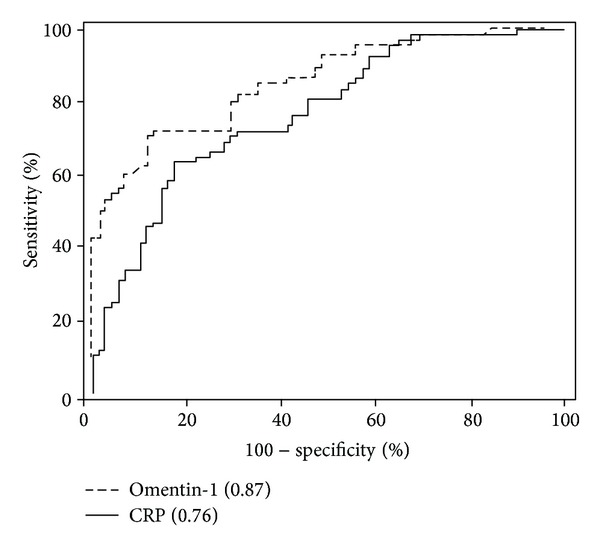
Serum omentin-1 levels as an activity index in Crohn's disease (CD) patients. The cutoff value of serum omentin-1 levels was 303.43 ng/mL (yielding sensitivity and specificity values of 74.5% and 84.0%). In contrast, when the cutoff value of CRP levels was set at 3.4 mg/mL, the sensitivity was 63.0% and the specificity was 72.3%. We found that the area under the ROC curve (AUC) for serum omentin-1 levels was 0.87, whereas the AUC for CRP levels was 0.76.

By generating a receiver operating characteristic (ROC) curve, the sensitivity and specificity of serum omentin-1 for remission and active CD by CDAI were determined (Figure 4). Cutoff points were determined by the maximum sum of sensitivity and specificity. The cutoff value of serum omentin-1 levels was 303.43 ng/mL (yielding sensitivity and specificity values of 74.5% and 84.0%). In contrast, when the cutoff value of CRP levels was set at 3.4 mg/mL, the sensitivity was 63.0% and the specificity was 72.3%. We found that the area under the ROC curve (AUC) for serum omentin-1 levels was 0.87, whereas the AUC for CRP levels was 0.76. These results emphasize the usefulness of monitoring serum omentin-1 levels for the evaluation of the disease activity of CD.

## 4. Discussion

In the present prospective study, we observed a significant decrease in serum omentin-1 levels in patients with active CD compared to patients with FGDs with no organic gastrointestinal pathology and healthy controls. In addition, expression of omentin-1 was decreased at mRNA and protein levels in inflamed colonic tissues in active CD than that in noninflamed colonic tissues. The correlation of serum omentin-1 with disease activity in CD is superior to that of CRP with disease activity. These results indicate that the already decreased omentin-1 levels are associated with inflammatory status in CD. Serum omentin-1 is thus a new biomarker for CD disease activity.

Inflammation has been considered as a critical player in the pathogenesis of CD [14]. Elevated levels of various circulating inflammatory markers, such as tumour necrosis factor alpha (TNF-*α*) and CRP have been suggested to be associated with CD [14, 15]. Omentin-1, one newly discovered adipokine, is shown to act as an anti-inflammatory mediator. Omentin can inhibit TNF-induced vascular inflammation in human endothelial cells [11]. Omentin was also reported to inhibit TNF-*α*-induced vascular cell adhesion molecule-1 expression via preventing the activation of p38 and c-Jun N-terminal kinase at least in part through the inhibition of superoxide production [16]. Our results show that serum and colonic expressions of omentin-1 in patients with active CD were downregulated, which supports the point that omentin may play an important anti-inflammatory role. Furthermore, serum omentin-1 was negatively correlated with TNF-*α* and CRP [17, 18]. Similarly, we also found in our study that serum omentin-1 levels were negatively correlated with CRP. Hence, decreased levels of serum omentin-1 may reflect decreased anti-inflammatory activity and then elevated levels of inflammatory markers in the development and progression of CD.

In sum, our study showed that serum and colonic omentin-1 expressions were decreased in active CD patients compared with controls. The correlation of serum omentin-1 with disease activity in CD is superior to that of CRP. Serum omentin-1 is thus a new biomarker for CD disease activity. These findings should be further validated in long-term and more prospective studies.

## Figures and Tables

**Table 1:** Patient characteristics.

Group	Active CD (*n* = 112)	CD in remission (*n* = 128)	FGDs (*n* = 120)
Gender ratio	0.50	0.53	0.57
Age (years)	34.51 ± 12.04	36.38 ± 13.92	39.05 ± 15.20
Weight (kg)	59.68 ± 13.24	61.83 ± 14.44	63.03 ± 14.51
Height (cm)	166.10 ± 9.06	164.37 ± 10.08	166.46 ± 9.28

**Table 2:** Disease distribution.

Distribution	Active CD	CD in remission
Isolated ileum	16	20
Isolated colon	22	16
Isolated ileocolonic	34	20
Isolated anal/perineal	6	26
Anal-perineal associated with another site	30	38
Ileal, colonic, or ileocolonic associated with another nonanal/perineal site	4	8
